# Maternal Risk of Breeding Failure Remained Low throughout the Demographic Transitions in Fertility and Age at First Reproduction in Finland

**DOI:** 10.1371/journal.pone.0034898

**Published:** 2012-04-18

**Authors:** Jianghua Liu, Anna Rotkirch, Virpi Lummaa

**Affiliations:** 1 Department of Animal & Plant Sciences, University of Sheffield, Sheffield, United Kingdom; 2 Oxford Centre for Population Studies, Department of Social Policy and Intervention, University of Oxford, Oxford, United Kingdom; 3 Wissenschaftskolleg zu Berlin – Institute for Advanced Study, Berlin, Germany; University of Debrecen, Hungary

## Abstract

Radical declines in fertility and postponement of first reproduction during the recent human demographic transitions have posed a challenge to interpreting human behaviour in evolutionary terms. This challenge has stemmed from insufficient evolutionary insight into individual reproductive decision-making and the rarity of datasets recording individual long-term reproductive success throughout the transitions. We use such data from about 2,000 Finnish mothers (first births: 1880s to 1970s) to show that changes in the maternal risk of breeding failure (no offspring raised to adulthood) underlay shifts in both fertility and first reproduction. With steady improvements in offspring survival, the expected fertility required to satisfy a low risk of breeding failure became lower and observed maternal fertility subsequently declined through an earlier age at last reproduction. Postponement of the age at first reproduction began when this risk approximated zero–even for mothers starting reproduction late. Interestingly, despite vastly differing fertility rates at different stages of the transitions, the number of offspring successfully raised to breeding per mother remained relatively constant over the period. Our results stress the importance of assessing the long-term success of reproductive strategies by including measures of offspring quality and suggest that avoidance of breeding failure may explain several key features of recent life-history shifts in industrialized societies.

## Introduction

With dramatic changes in the socio-economic environments of many human populations over the past 200 years, there have been intriguing shifts in maternal life-history traits including a decline in fertility and a postponement of age at first reproduction. Demographers define the shift of fertility from a high level (typically more than five children per mother) to a low one (less than three children per mother) as a key feature of the first demographic transition [1], [2]. The persistent postponement of first reproduction from an early age (typically not later than 25 years) to a later one (around 30 years now in many European countries such as Germany and Spain [3]) is defined as a key feature of the second demographic transition [2], [4]. The postponement of first reproduction is often accompanied by a fertility level lower than two children per woman, i.e. a fertility rate below population replacement level [2]. For most European countries, the first demographic transition started in the latter half of the 19th century and lasted until around 1970 [1], [5], when the second transition started [2]. Both the transition in fertility and that in age at first reproduction took place in a context of increased resources available for reproduction as provided by the increase in the standard of living [1].

From an evolutionary perspective, at first glance the fertility decline is puzzling: Animals are expected to reproduce more when resources become more abundant (for the example of birds, see [6], [7]; for the example of mammals, see [8], [9]), so fertility should have increased rather than decreased with the economic prosperity in developed European countries. Various explanations from different evolutionary perspectives have been raised to address this paradox (reviewed first in [10]). Firstly, studies on agro-pastoral societies in Kenya in the 1980s and 1990s suggest that increasing proportion of extra-somatic resources (e.g. herds or lands in these societies or other resources in modern societies) engage parents in a new form of offspring quantity-quality trade-off in which offspring mating and reproductive success is determined by the quantity of wealth that they inherited. Consequently, if parents wish to maximize their reproductive success, they must optimize inherited wealth among offspring by producing a smaller family size [11], [12]. Secondly, based on an empirical analysis of the fertility behaviour of men in the American city of Albuquerque, New Mexico, in the 1990s, it is concluded that parents in competitive societies appear to be driven by the evolved psychology to invest in their offspring so as to increase their own and their offspring's socio-economic status, which would be translated into mating and reproductive success in the environment of evolutionary adaptedness of that psychology [13]. In modern societies, socio-economic status or its major component income is strongly correlated with individual education; consequently, parents now focus limited resources (including time) on fewer children to improve offspring education and skill acquisition [13]. Finally, others have proposed that one of the causes of the fertility decline could be the asymmetrical transmission of cultural norms, i.e. those professionals with a smaller family size have a larger influence on transmitting cultural norms [14]. Some empirical evidence supports this idea (see [14] for specific examples); for example, in modern societies, those with a larger family size have reduced upward mobility in social status, which consequently limits their role in spreading their own family norms, i.e. a large family size.

Although these studies have contributed to our evolutionary understanding of fertility declines, they have some limitations. Firstly, data used in such studies are typically restricted to a cross-section rather than spanning the whole course of the first demographic transition; however, the evolutionary implications of changing fertility cannot be fully understood without comparing the long-term reproductive consequences of fertility rates at different stages of the transition. Secondly, such evolutionary analyses have paid relatively little attention to the postponement of first reproduction after maturity (for the evolution of maturity *per se*, see [15], [16]), a change that was not concomitant with the fertility decline [17], but started only at the end of it. As the decline in fertility, the postponement of first reproduction is also evolutionarily puzzling in view of earlier sexual maturity and thus increased reproductive opportunity due to improved nutrition and juvenile survival in modern societies [18].

There are several possibilities as to why women might postpone their first reproduction in the second demographic transition. One is that increasing the socio-economic status of future offspring may require parents to postpone reproduction and, for instance, to invest in their own education as a way of accumulating resources [13]. However, this does not explain why the postponement of first reproduction did not occur simultaneously with the fertility decline during the first demographic transition, when postponement could have served the dual functions of accumulating resources and limiting fertility. Furthermore, a later age at first birth in modern societies does not seem to improve offspring reproductive performance [19], although possible time trends in such effects have not been investigated. Another possibility is that improvements in life expectancy might lead to a postponement of first reproduction. Both cross-cultural and within-country studies indicate that in equilibrium conditions, females reproduce early when adult life-expectancy is low [20]–[24]. However, these studies have ignored reproduction after the first birth. If lifetime fertility is considered, a third possibility arises: Improved offspring survival may reduce the necessity to start reproduction early in order to be able to compensate for expected offspring deaths (e.g. [25]). Reduction of the uncertainty regarding offspring survival and the subsequent risk of failing to raise any produced offspring to adulthood may thus be relevant to both the decline in fertility [26]–[28] and the postponement of first reproduction during the historical demographic transitions (for the link between a high risk and early reproduction, see [20]). However, this hypothesis has not yet been tested empirically.

In this study, we use data from succeeding generations of Finnish mothers reproducing across the whole period of the first demographic transition (1880s–1960s) and the beginning of the second transition (1970s) to investigate whether and how individual reproductive behaviours responded to changes in maternal risk or probability of breeding failure. We define breeding failure as failing to raise at least one of the produced offspring to adulthood (see also [29]). Specifically, we analyze how this risk was associated with shifting fertility and age at first reproduction during a century of demographic change. Since both offspring quantity (i.e. maternal lifetime fertility) and survival contribute to quantifying the maternal risk of breeding failure (see methods), we also investigate dynamic associations between fertility/age at first reproduction and offspring survival across the period. Additionally, we investigate the consequences of the shifts in life-history traits for maternal reproductive success, measured as the number of breeding offspring (see [30]). Finally, we investigate how differences in resources, measured in terms of socio-economic status, affected the above associations and how this impact changed with time.

## Materials and Methods

### Study populations

We investigate life-history shifts during the recent demographic transitions using records of succeeding generations of mothers living in three parishes of Finland. The Lutheran Church has kept census, birth/baptism, marriage and death/burial registers of each parish in the country since the 17th century, covering nearly the whole population of Finland from 1749 onwards. A sample of maternal and paternal pedigrees (lineages) from 1749 until the modern day (max. 10 generations) has been reconstructed from the original archives of local Lutheran churches by professional genealogists and for the recent cohorts, also from the published genealogies available in public libraries (e.g. [31]–[34]) that use the same original sources of data. The records included information on birth and death, marriage and reproduction (if any), and immigrations and emigrations (if any). Additionally, there were occupation records of the husband of each family, basing upon which we classify maternal socio-economic status into three categories (upper class, middle class and lower class; see also [35]) after taking into account the changing levels of relative income of different occupations across time. Our use of the church records is conducted in line with Finnish legislation and ethical guidelines of the University of Sheffield. Specifically, the original and microfilmed copies of the church records are now maintained in local Population Archives and they are freely available for the public e.g. for genealogical research. The dataset using these public archives and published genealogies is set up using ID codes rather than names, and no personal data are recorded in addition to the birth, marriage, childbirth and death dates.

Geographically, two of the parishes are located in the south-western archipelago and one is located in the Finnish mainland (archipelago: Hiittinen 60°N, 22°30′E, Kustavi 60°30′N, 21°30′E; mainland: Ikaalinen 61°45′N, 23°E) [36], [37]. Although all the base individuals in the pedigrees from 1749 onwards as well as the majority of their descendants resided in these parishes, our dataset also includes most pedigree members who migrated within Finland.

In temporal scale, we use records of mothers who gave their birth to first child between 1880 and 1979 (100 years; 1947 mothers in total, including those who died at first birth and whose baby also died); for life-history patterns in the population before the first demographic transition, see [38]. Our study covers the entire first demographic transition in Finland and the start of the second one in the 1970s [1], [39], enabling analyses of the dynamic associations between key life-history traits at different phases of the transitions. The transformation from an agrarian to an industrial country began in Finland in the 1880s [40] and it was only from that decade onwards that significant changes appeared in terms of food supply, health care, the proportion of the non-agricultural population, life expectancy at birth, income (e.g. gross domestic product per capita) and female education level–changes considered relevant to shifts in life-history traits in Finnish females [39]. Mothers starting reproduction after 1980 are not included in this study, since for most of them, full information on their offspring survival and breeding is not yet available and additionally, for some of them, their fertility years are not yet over.

The relevant life history traits of the mothers in this study are summarized in Table 1. It is worth noting that due to regional differences in fertility, the studied mothers had lower fertility and more advanced ages at first birth than the general levels across Finland, but the time trends of the two traits among such mothers were consistent with the trends holding for the entire country [41], [42].

**Table 1:** Descriptive statistics of relevant life history traits along time.

Decades	Sample	M-AFR	M-ALR	M-span	M-BI	Quantity	Survival	Breeding	M-LRS
1880s	291	26.6±0.3	38.0±0.4	11.4±0.4	2.3±0.1	5.3±0.2	0.73±0.03	0.37±0.03	2.0±0.1
1890s	271	25.1±0.3	36.0±0.4	10.9±0.4	2.3±0.1	5.0±0.2	0.75±0.03	0.41±0.03	2.2±0.2
1900s	246	25.9±0.3	35.9±0.4	10.1±0.5	2.3±0.1	4.6±0.2	0.83±0.02	0.50±0.04	2.3±0.1
1910s	195	25.8±0.4	35.3±0.5	9.5±0.5	2.3±0.1	4.5±0.2	0.85±0.03	0.58±0.04	2.4±0.2
1920s	266	25.7±0.3	33.8±0.4	8.1±0.4	2.6±0.1	3.5±0.1	0.89±0.02	0.67±0.03	1.9±0.1
1930s	257	26.2±0.3	33.4±0.4	7.2±0.4	2.5±0.1	3.2±0.1	0.93±0.02	0.65±0.04	1.9±0.1
1940s	173	28.7±0.4	33.8±0.5	5.1±0.4	2.2±0.1	2.6±0.1	0.93±0.02	0.56±0.06	1.6±0.2
1950s	78	25.7±0.6	30.7±0.6	5.0±0.6	2.3±0.2	2.4±0.2	0.99±0.01	0.83±0.05	2.0±0.2
1960s	79	23.7±0.4	28.7±0.5	5.0±0.5	2.4±0.2	2.3±.1	0.99±0.01	0.93±0.04	2.0±0.1
1970s	91	25.6±0.5	30.1±0.6	4.5±0.5	2.4±0.2	2.1±0.1	0.99±0.01	0.96±0.04	1.8±0.2
Total Mean		26.0±0.1	34.6±0.2	8.6±0.2	2.4±0.0	3.9±0.1	0.86±0.01	0.56±0.01	2.1±0.1
Correlation		*ρ* = 0.008	*ρ* = −0.34	*ρ* = −0.34	*ρ* = 0.033	*ρ* = −0.39	*ρ* = 0.35	*ρ* = 0.37	*ρ* = −0.005
		*t* = 0.36	*t* = −16.00	*t* = −15.87	*t* = 1.33	*t* = −18.71	*t* = 15.12	*t* = 10.06	*t* = −0.12
		*df* = 1945	*df* = 1945	*df* = 1945	*df* = 1598	*df* = 1945	*df* = 1684	*df* = 655	*df* = 655
		*P* = 0.36	*P*<0.001	*P*<0.001	*P* = 0.18	*P*<0.001	*P*<0.001	*P*<0.001	*P* = 0.90

Note. Sample, sample size; Decade, the decade when mothers gave their first births; M-AFR, maternal age at first reproduction; M-ALR, maternal age at last reproduction; M-span, maternal reproductive lifespan; M-BI, maternal birth interval (calculated by considering only the cases where reproductive span was larger than zero); Quantity, offspring quantity or maternal lifetime fertility; Survival, offspring survival rate at age 15; Breeding, offspring breeding probability; M-LRS, maternal lifetime reproductive success (calculated using the algorithm mentioned in section **2.2.**); Correlation, the correlation between the specific traits (M-AFR, M-ALR, etc.) and the year when giving the first birth and 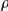 is sample estimate of Pearson's correlation coefficient; for weighted traits (offspring survival rate and breeding probability, and maternal lifetime reproductive success), mean and standard error are calculated using weighted formulas (code available from authors); for weighted traits (offspring survival rate and breeding probability, and maternal lifetime reproductive success), correlation coefficients are calculated using only the records of mothers with weight as 1.

### Statistical methods

Along the line of [38], maternal risk of breeding failure can be quantified approximately as 
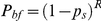
; here, 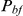 is maternal risk or probability of breeding failure, 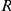 is offspring quantity and 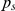 is offspring survival rate at adulthood (age 15 in this study). Thus the determinants of maternal breeding failure risk are offspring quantity and offspring survival rate. As mentioned, the determinants of reproductive success are offspring quantity and offspring breeding probability (see also [43]). Consequently, to study how fertility and age at first reproduction were associated dynamically with maternal risk of breeding failure, reproductive success and their determinants over time, we analyze: (A) the dynamic association between fertility and age at first reproduction, as well as the dynamic associations between fertility/age at first reproduction and (B) offspring survival rate at age 15; (C) offspring breeding probability; (D) maternal lifetime reproductive success; (E) maternal risk of breeding failure (none of the produced offspring were raised to age 15) across the 100-year study period. Mothers giving first birth in the 1960s and 1970s with still incomplete offspring reproductive data are omitted from the analyses concerning offspring breeding traits (offspring breeding and maternal reproductive success).

We first use non-parametric Generalized Additive Models (GAMs) to capture plausible shapes of the above associations [44]. For instance, if a GAM suggests an association concerning fertility or age at first reproduction to be linear, we then include a linear term of fertility or age at first reproduction as a fixed effect in parametric mixed effect models (Generalized Linear Mixed Models, GLMM). In such GLMMs, parish (three levels) is included as a random effect to account for geographical differences, maternal socio-economic status (three levels) as a fixed effect to control for maternal resource availability, and the decade when a mother gave her first birth (10 levels) as a fixed effect to investigate how the associations varied with time. Since offspring quality in terms of survival and breeding was strongly associated with the period after their birth, we prefer a period analysis (first birth decade) to a maternal-birth-cohort analysis. In the GLMMs, maximum models are introduced firstly by including fertility or age at first reproduction (and, if needed, their quadratic polynomials where the variables are centred by subtracting the overall mean), decade, maternal socio-economic status and all of their possible interactions. Then, using a backward stepwise regression based on likelihood ratio test [45], a minimum adequate model is obtained from a maximum model. The test statistic is asymptotically chi-square distributed and the degrees of freedom correspond to the difference in the numbers of parameters in the models being compared. We use this procedure to test all interactions and the results are presented in Table S1. We also use it to remove those non-significant main effects not involved in significant interactions. During the model simplification, we find that middle and upper classes can be combined into one class whenever the socio-economic status was significant. Thus, in the final minimum adequate models, the socio-economic status is classified into non-lower class (including middle and upper classes; 1378 mothers in total) with stable income and lower class (569 mothers in total) with no stable income.

Of the response variables, fertility is modelled as a Poisson variable (only in studying its association with age at first reproduction), offspring survival rate at age 15 or breeding probability as binomial variables, and maternal breeding failure as a binary variable (no produced offspring surviving to age 15 versus at least one produced offspring surviving to age 15). In the GLMMs concerning both the Poisson variable and binomial variables, the coefficients are asymptotically normally distributed and the significance results from testing the coefficients of the main effects are given by a *z*-test (the default algorithm of lme4 in R, the statistical package we use in GLMMs).

Lifetime reproductive success is determined as the product of offspring quantity and the breeding probability among those offspring with a definite breeding census. For example, imagine the case where a mother had four offspring, among whom two were successfully tracked along their entire life to determine their breeding status, and among these tracked two, one bred (had at least one child) and the other failed to do so. For such mothers, we assign all four offspring a 50% breeding probability and the mother's lifetime reproductive success is determined as two breeding offspring. Reproductive success calculated in this way may not be an integer (count data) and is therefore Box-Cox transformed [44] and modelled as a normally distributed variable; relevant significance results are given by a *t*-test (the default algorithm from lme4). Using survival or breeding probability among tracked offspring to represent that among all offspring may cause bias, and so weighted regressions are used to downplay the influence of those mothers lacking complete survival or breeding information on all offspring. The weight corresponds to the proportion of offspring with survival information at age 15 in analyzing offspring survival rate, the proportion of offspring with information on breeding in analyzing offspring breeding probability and maternal reproductive success, and 1 or the proportion of offspring with survival information at age 15 according to whether a mother had at least one surviving offspring or not in analyzing maternal risk of breeding failure. However, the main results do not qualitatively change if we limit our sample to those families with full records for all offspring so that weighted regressions are not needed. The completeness of the data for corresponding response variables is listed in Table 2.

**Table 2:** The degrees of data completeness for the response variables investigated.

	Proportion of data with different degrees of completeness			
Variables	Complete (100%)	Incomplete (>0%)	Missing (0%)	Total
M-fertility	100%	0	0	100%
O-survival	86.60%	11.45%	1.95%	100%
O-breeding	33.74%	45.05%	21.21%	100%
M-LRS	33.74%	45.05%	21.21%	100%
M-RBF	96.66%	1.39%	1.95%	100%

Note. Complete–the variable value can be accurately determined (e.g. for 86.60% of the mothers under study, survival status (survival versus death) at age 15 of all produced offspring can be accurately determined); Incomplete–the variable value was estimated using the records available for some of all offspring (e.g. for 11.45% of the mothers, survival status data were available for some (at least one, but not all) of their offspring); Missing–there was no way to estimate the variable value and relevant mothers must be excluded from the analyses (e.g. for 1.95% of the mothers, survival status data were missing for all of their offspring); M-fertility–maternal lifetime fertility; O-survival–offspring survival rate at age 15; O-breeding–offspring breeding probability; M-LRS–maternal lifetime reproductive success; M-RBF–maternal risk of breeding failure.

All statistical analyses are carried out in R 2.11.1 [46] using statistical packages “lme4” for GLMM [47], “mgcv” for GAM [48] and “lattice” for plotting [49]. All statistical results correspond to transformed scales, e.g. logit scale when modelling a binomial variable, but the plots correspond to raw or back-transformed scale.

## Results

### Fertility and age at first reproduction

Offspring quantity declined with time: In the 1880s, the average number of offspring per mother was over five, but in the 1970s, it was just around two (Table 1). Maternal ages at first reproduction showed no clear directional time trends and averaged 26.02 years across the 100-year study period (Table 1). Age at first reproduction was however slightly U-shaped with the lowest value in the 1960s, in agreement with national census data from the Council of Europe [41] indicating that as a whole the postponement of first births started around 1970 in Finland.

**Figure 1:**
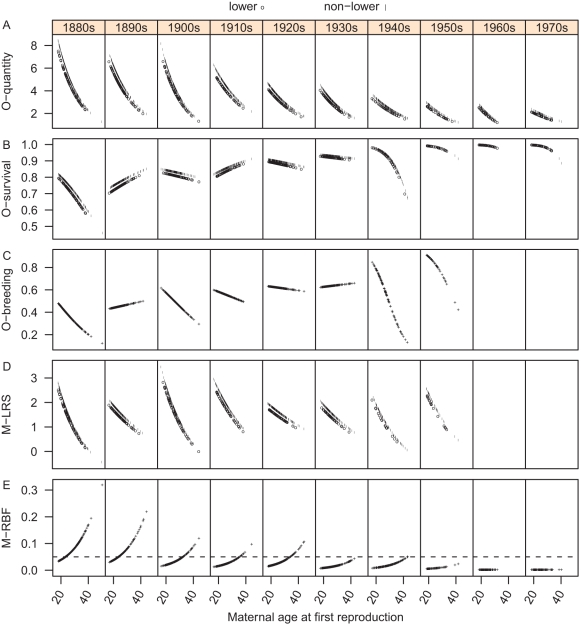
Changes in the studied traits in response to maternal age at first reproduction from the 1880s to 1970s. A, offspring quantity (O-quantity); B, offspring survival rate at age 15 (O-survival); C, offspring breeding probability (O-breeding); D, maternal reproductive success (M-LRS); E, maternal risk of breeding failure (M-RBF). Time/decade is shown at the top. When there was a significant difference between the social classes, “o” denotes lower class mothers and “|” denotes non-lower (middle or upper) class mothers; when there was no difference, only one symbol (+) is used. Offspring breeding probability and maternal reproductive success data are not yet available for mothers giving first birth in the 1960s and 1970s.

In each decade, offspring quantity decreased with a postponement in maternal age at first reproduction (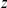 = −11.54, 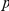
<0.001). However, the magnitude of the negative association declined with time (Fig. 1A; Table S1), reflected in the decreased range/standard errors of offspring quantity with time (Table 1). In the post-war decades, even a mother who began reproduction early might still have ended up with only two offspring (Fig. 1A; Table 1). Later age at first reproduction meant lower fertility for both lower class and non-lower class mothers (Table S1); however, given the same age at first reproduction, middle or upper class mothers had on average more offspring than lower class mothers (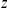 = 4.21, 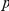
<0.001) across the whole period under study (Fig. 1A; Table S1).

### Fertility/age at first reproduction and offspring survival rate at age 15

**Figure 2:**
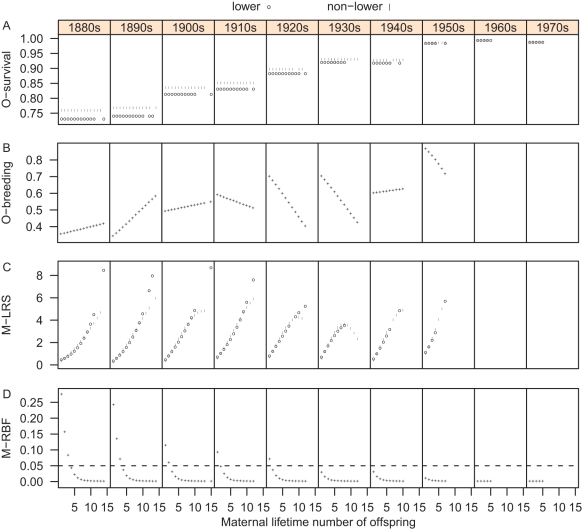
Changes in the studied traits in response to maternal fertility from the 1880s to the 1970s. A, offspring survival rate at age 15 (O-survival); B, offspring breeding probability (O-breeding); C, maternal reproductive success (M-LRS); D, maternal risk of breeding failure (M-RBF). Time/decade is shown at the top. Abscissa of each discrete point represents an integer number of children, i.e. maternal fertility. When there was a significant difference between the social classes, “o” denotes lower class mothers and “|” denotes non-lower (middle or upper) class mothers; when there was no difference, only one symbol (+) is used. Offspring breeding probability and maternal reproductive success data are not yet available for mothers giving first birth in the 1960s and 1970s.

Offspring survival rate improved with time: In the 1880s, on average 75% of the children born in that decade survived to age 15, whereas from the 1950s onwards close to 100% survived (Fig. 2A; Fig. 1B; Table 1).

At an individual mother level, there was no significant association between fertility and offspring survival rate, and this result did not vary across time or between socio-economic groups (Fig. 2A; Table S1). When controlling for maternal fertility, offspring born to middle or upper class mothers had a higher chance to survive to age 15 than those born to lower class mothers (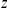 = 2.05, 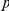
<0.05) across the whole period (Fig. 2A; Table S1).

A delayed maternal age at first reproduction was not statistically significantly associated with a lower offspring survival rate (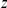 = −1.79, 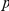 = 0.07) for either socio-economic groups. The association was negative in most decades but varied with time and turned positive in two decades (1890s and 1910s) (Fig. 1B; Table S1). When controlling for maternal age at first reproduction, offspring born to non-lower class mothers had a higher chance of surviving to age 15 than those born to lower class mothers (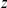 = 2.00, 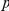
<0.05) across time (Fig. 1B; Table S1).

### Fertility/age at first reproduction and offspring breeding probability

Offspring breeding probability improved with time. However, it did not approximate 100% even in the 1950s when offspring survival rate came close to 100% (Fig. 2B; Fig. 1C). In other words, not every surviving offspring bred: On average across the study period, 25% females surviving to age 45 never had children of their own (calculated by assigning childless females a hypothetical “first birth year” as their birth year plus 26 years; see Methods).

The overall association between maternal fertility and offspring breeding probability was not significant (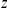 = −1.02, 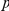 = 0.31) in any socioeconomic group; however, this association varied with time, being positive in some decades and negative in others (Fig. 2B; Table S1). When controlling for maternal fertility, the breeding probability of offspring born to middle or upper class mothers was not significantly different from that of those born to lower class mothers across the whole period (Table S1).

As a whole, delayed maternal age at first reproduction was associated with reduced offspring breeding probability (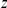 = −4.54, 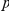
<0.001) for both socioeconomic groups (Table S1). However, their association varied across time and turned slightly positive in some decades (1890s and 1930s) (Fig. 1C; Table S1). When controlling for maternal age at first reproduction, the effect of maternal socio-economic status on offspring breeding probability was not significant across the ten decades (Table S1).

### Fertility/age at first reproduction and lifetime reproductive success

Lifetime reproductive success remained relatively constant over the 100 years under study, with an overall average at two breeding offspring per mother (Table 1; Fig. 2C; Fig. 1D).

On the whole, reproductive success was associated positively with fertility (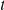 = 12.19, 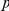
<0.001): For most levels of fertility (<10) the association was approximately linearly positive; however, there was an interaction between the quadratic term of fertility and time (e.g. reproductive success decreased with an increase in fertility when fertility was high in some decades) (Fig. 2C; Table S1). A quadratic interaction with socio-economic status was also significant; e.g. when fertility was high, the curved response of reproductive success to fertility differed between mothers from different socio-economic groups (Fig. 2C; Table S1). However, in general, mothers from two socio-economic groups had similar reproductive success under the same fertility (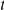 = 0.09, 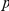 = 0.93) across the study period (Table S1).

In all decades, maternal lifetime reproductive success was negatively associated with age at first reproduction (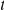 = −8.65, 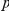
<0.001) for both socio-economic groups (Fig. 1D; Table S1). However, the strength of this association varied across time with reproductive success being reduced more in some decades and less in others with the same delays in age at first reproduction (Fig. 1D; Table S1). With the same age at first reproduction, non-lower class mothers had higher reproductive success across the ten decades (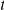 = 1.78, 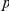 = 0.08; although the 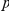-value is larger than 0.05, likelihood ratio test suggested to retain maternal socio-economic status in the minimum model) (Fig. 1D; Table S1).

### Fertility/age at first reproduction and maternal risk of breeding failure

Maternal risk of breeding failure declined with time (Fig. 2D; Fig. 1E). In the 1880s about 7% of mothers failed to raise any of their produced offspring to age 15 (weighted proportion from raw data, with weight being the proportion of offspring whose survival status was determined). In the 1930s and 1940s, maternal risk of breeding failure became relatively low and was under 5% even if a mother produced a sole offspring. Since the 1950s the risk of breeding failure was almost zero.

Lower fertility was associated with an increased risk of breeding failure (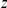 = −6.76, 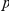
<0.001) for mothers in both socio-economic groups in all decades across the period under study (Table S1). When controlling for fertility, maternal risk of breeding failure declined with time and became very low even for extremely small family sizes towards the end of the study period (Fig. 2D). Additionally, when controlling for fertility, maternal risk of breeding failure did not differ between lower class and non-lower class mothers across the whole period (Table S1).

Maternal risk of breeding failure increased with a postponement of maternal age at first reproduction (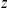 = 3.68, 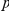
<0.001) for both socio-economic groups in all decades across the study period (Fig. 1E; Table S1). As in the case of fertility, maternal risk of breeding failure declined with time when controlling for age at first reproduction (Table S1). Towards the end of the study period, given that the risk of breeding failure was extremely low even for mothers producing only one offspring (see the paragraph above), a positive association between age at first reproduction and the risk cannot be observed; and even when a mother postponed her first reproduction until age 35, the maternal risk of breeding failure was clearly below 5% (Fig. 1E). When controlling for age at first reproduction, maternal risk of breeding failure did not differ between lower class and non-lower class mothers across the time period (Table S1).

## Discussion

The fertility decline and postponement of first birth (i.e. later age at first reproduction) in industrialized societies during the past 200 years are evolutionarily puzzling, because animals are expected to reproduce more and earlier when resources grow more abundant [6]–[9]. Previous studies have suggested that a reduction of uncertainty in offspring survival and subsequently, in the risk of failing to raise at least one of the produced offspring to adulthood (maternal breeding failure) might have promoted life-history shifts during the fertility transition [26]–[28]. However, this hypothesis has not yet been substantiated empirically, partly owing to the rarity of individual-based data across time, and has not been linked to the postponement of age at first reproduction. Our study is the first to investigate empirically the dynamic associations between the risk of breeding failure and the key life-history traits that shifted during the demographic transitions, using life-history records of the historical Finnish mothers whose first reproduction spanned the whole course of the first demographic transition (1880s–1960s) and the beginning of the second one (1970s) in Finland.

Our results show that offspring quantity did not compromise offspring survival rate or breeding probability at an individual mother level over the 100 years of radical fertility decline in Finland (for a similar result from the United States, see [19]). In each decade, a mother's risk of breeding failure increased and her reproductive success decreased when fertility was lower. On the whole, delayed maternal age at first reproduction was associated negatively with both offspring quantity and survival rate, like the case with the Finnish women from a period before the demographic transitions [38]. Such associations may not necessarily be causal, given the possibility of a correlation between early reproduction and phenotypic quality (see also [38]). We also find that on the whole, delayed first reproduction was associated with a lower offspring breeding probability. Due to such associations with offspring quantity and quality, later age at first reproduction was associated with a marked decline in reproductive success and an increase in the risk of breeding failure.

Interestingly, in each decade of our study period, the risk of breeding failure corresponding to the average level of fertility in that decade was around or below 5% (e.g. in the 1880s, the risk corresponding to the average fertility level of 5.3 children was about 2%) (Table 1; Fig. 2D). In each of the early decades, the required fertility satisfying a 5% breeding failure risk was close to the observed mean fertility. With improvement in offspring survival rate, the required fertility level became lower, and it was following this that the actual fertility declined. However, there was a time lag: A decline in required fertility preceded that in observed fertility and, as a result, the observed mean fertility was higher than the required one in each decade. For example, the required fertility declined from four children in the 1880s to three children in the 1900s (Fig. 2D), while the observed average fertility declined to three children only in the 1930s (Table 1). This decline was mainly driven by younger ages at last reproduction (Pearson's coefficient of correlation between age at last reproduction and fertility: 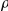 = 0.62, *t*_1945_ = 34.77, *P*<0.001) and thus a contraction of the total reproductive span (Pearson's coefficient of correlation between the span and fertility: 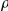 = 0.85, *t*_1945_ = 70.66, *P*<0.001) in a context of relatively constant age at first reproduction and birth intervals across time (Table 1). Presumably, a mother stopped reproduction earlier once observing, firstly, that the offspring already produced survived well and, secondly, that there was an evident decline in mortality among younger generations compared with the childhood of the mother's own cohort [27], [28]. This finding is consistent with classic demographic transition theory proposing that fertility decline typically follows a steady decline in offspring mortality [27]. However, in previous studies it has not been demonstrated empirically that fertility among mothers never declined to a level entailing a high risk of breeding failure. Our findings indicate a correspondence between fertility rates and a low risk of breeding failure rather than a correspondence between fertility rates and mortality rates; therefore, we do not suggest here that a decline in mortality would necessarily precede a decline in fertility in every population experiencing fertility decline (see [50]).

Given that postponing first reproduction is also effective in limiting fertility [51], it is puzzling why the fertility decline occurred by means of ceasing reproduction earlier, rather than by starting reproduction at a later age. This question has not attracted much attention, and two observations are of interest here. Firstly, in each decade the average age at first reproduction did not entail a high risk of breeding failure (Fig. 1E) – just as the case with fertility. Secondly, a steady postponement of first reproduction was first manifest in Finland from about 1970 [2], a time when female life expectancy had increased significantly [52] and the risk of breeding failure at any age at first reproduction was close to zero (Fig. 1E). Few previous studies have reported comparable analyses; one of the existing studies on a U.S. sample used a lineage success index different from ours [53], and thus their results cannot be directly compared to ours. Our results indicate that it has been “safe” to moderately postpone first reproduction since the 1970s, the beginning of the second demographic transition. However, excessive postponement (e.g. past age of 35 years) might well result in childlessness (e.g. [53]), the rate of which was high in historical Finland and has increased somewhat during the second transition [2]. In view of these two observations, the reasons why the postponement of first births was used more conservatively than ceasing reproduction at an earlier age in limiting fertility can be inferred as follows. Firstly, the accumulated risk of dying before any reproduction always increases with age [54]. Despite a persistent increase (typical in non-equilibrium conditions; see [22], [55]), female life expectancy at birth was shorter than 60 years during most of our study period [52]. Secondly, there is no way to observe the survival of one's own offspring before first reproduction and compared with the age at last reproduction, the age at first reproduction always means more uncertainty with respect to breeding failure.

Our results on the associations between maternal risk of breeding failure and maternal fertility or age at first reproduction during the Finnish demographic transitions thus suggest some inclination to avoid breeding failure among human females, a suggestion also made from some other studies. For example, interviews with contemporary African American teenage mothers-to-be indicate that few want to die without leaving any surviving offspring [21]. This inclination is also considered as responsible for the risky sexual behaviour among girls in sub-Saharan Africa where adult lifespan is short and infant mortality is high [20] (see also [23]). Currently, the proximate mechanisms channelling the avoidance of breeding failure are unclear and merit further exploration.

Traditionally, demographic studies have focused on broad changes in mortality and fertility but have paid little attention to mother-specific numbers of offspring recruited to the breeding population. We find that average maternal reproductive success within the populations under study remained relatively constant at two breeding offspring across the 100 years covering both the first demographic transition and the beginning of the second one. In other words, from an evolutionary perspective, a female starting reproduction in the 1950s was just as ‘fit’ as a female in the 1880s, although she reproduced much less. Such a pattern emerged despite drastic changes in the population fertility rates, because fertility (offspring quantity) and offspring survival rate and breeding probability (offspring quality) shifted in opposite directions, and the reduction in quantity was compensated by the improvement in quality. If maternal fertility continues to decline below the level of two offspring, such a compensation cannot continue even if offspring breeding probability might reach 100%, which was actually never achieved in our study period.

On the whole, our main findings were not significantly modified by resource levels, i.e. the associations found in this study were similar for mothers in all social classes. However, socio-economic differences did affect both offspring quantity and offspring quality in terms of survival rates. Firstly, a mother in the middle or upper classes had on average more offspring than a mother in the lowest social class across the whole period when controlling for other factors. This finding is in contrast to the standard claim that the first demographic transition reversed the correlation between fertility and wealth found in earlier agrarian societies (e.g. [10]). A positive correlation between socio-economic status and fertility may still be observed once a proper contrast in socio-economic groups is made (e.g. middle or upper classes vs. lower classes) and attention is focused on homogeneous sub-populations (e.g. parish populations in this study; see [56]). Secondly, offspring survival rate was associated positively with socio-economic status, which was consistent with the prediction about parental investment in modern societies [13]. However, the breeding success of offspring was unrelated to their parental socio-economic status. Offspring born to lower class mothers may have developed reproductive strategies different from those born to higher class mothers in order to help them gain similar breeding success [23], [57]. This finding suggests that the evolutionary importance (i.e. the contribution to offspring breeding) of ensuring wealth transmissions or socio-economic success of offspring may be more limited than has been suggested (e.g. [58]), at least in relatively homogenous industrialised populations.

Our study investigates key life-history traits in the same populations across the demographic transitions using individual-based data on maternal reproductive behaviour. Most importantly it investigates all these with respect to the long-term consequences, i.e. breeding failure and reproductive success, of the changing strategies. This approach contributes to our evolutionary understanding of the recent radical shifts of life-history traits in demographic transitions. But in order to obtain a general picture, in the future it will be necessary to analyze relevant associations in other populations. While our study only focuses on mothers, also of interest might be to consider both sexes as well as individuals who for one or another reason do not reproduce at all.

## Supporting Information

Table S1 **Likelihood-ratio test of the main and interaction effects in maximum models in analyzing specific associations.**
(DOC)
