# Supplementary material for: Maternal Risk of Breeding Failure Remained Low throughout the Demographic Transitions in Fertility and Age at First Reproduction in Finland
Source: PLoS One. 2012 Apr 18;7(4):e34898. doi: 10.1371/journal.pone.0034898 (PMC3329549; doi:10.1371/journal.pone.0034898)
Supplement: Table S1 — Likelihood-ratio test of the main and interaction effects in maximum models in analyzing specific associations. (DOC) [file pone.0034898.s001.doc]

Table S1. Likelihood-ratio test of the main and interaction effects in maximum models in analyzing specific associations

|  |  |  | Test results | | |
| --- | --- | --- | --- | --- | --- |
| Association between | Interaction effects | Hypothesis tested | *df* | Chi-square | *P*-value |
| M-fertility and M-AFR | M-AFR : decade | The association between M-fertility and M-AFR did not change with time | 9 | 18.05 | <0.05 |
|  | M-AFR : M-SES | The association between M-fertility and M-AFR was independent of SES | 1 | 0.65 | 0.42 |
|  | M-SES : decade | The effect of M-SES on lifetime fertility did not change with time | 9 | 14.32 | 0.11 |
| O-survival and M-fertility | M-fertility : decade | The association between O-survival and M-fertility did not change with time | 9 | 8.46 | 0.49 |
|  | M-fertility : M-SES | The association between O-survival and M-fertility was independent of M-SES | 1 | 0.47 | 0.49 |
|  | M-SES : decade | The effect of M-SES on O-survival did not change with time | 9 | 6.55 | 0.68 |
|  | M-fertility | O-survival was not linked with M-fertility when controlling for M-SES and decade | 1 | 0.87 | 0.35 |
| O-survival and M-AFR | M-AFR : decade | The association between O-survival and M-AFR did not change with time | 9 | 25.08 | <0.01 |
|  | M-AFR : M-SES | The association between O-survival and M-AFR was independent of M-SES | 1 | 2.06 | 0.15 |
|  | M-SES : decade | The effect of M-SES on O-survival did not change with time | 9 | 5.90 | 0.75 |
| O-breeding and M-fertility | M-fertility : decade | The association between O-survival and M-fertility did not change with time | 7 | 23.28 | <0.01 |
|  | M-fertility : M-SES | The association between O-breeding and M-fertility was independent of M-SES | 1 | 0.30 | 0.58 |
|  | M-SES : decade | The effect of M-SES on O-breeding did not change with time | 7 | 12.78 | 0.08 |
|  | M-SES | O-breeding did not depend on M-SES when controlling for M-fertility and decade | 1 | 0.26 | 0.61 |
| O-breeding and M-AFR | M-AFR : decade | The association between O-breeding and M-AFR did not change with time | 7 | 24.42 | <0.001 |
|  | M-AFR : M-SES | The association between O-breeding and M-AFR was independent of M-SES | 1 | 0.50 | 0.48 |
| M-LRS and M-fertility | M-fertility^2 : decade | The association between M-LRS and M-fertility^2 did not change with time | 7 | 16.51 | <0.05 |
|  | M-fertility^2 : M-SES | The association between M-LRS and M-fertility^2 was independent of M-SES | 1 | 7.63 | <0.01 |
|  | M-SES : decade | The effect of M-SES on M-LRS did not change with time | 7 | 10.90 | 0.14 |
| M-LRS and M-AFR | M-AFR : decade | The association between M-LRS and M-AFR did not change with time | 7 | 21.57 | <0.01 |
|  | M-AFR : M-SES | The association between M-LRS and M-AFR was independent of M-SES | 1 | 0.17 | 0.68 |
|  | M-SES : decade | The effect of M-SES on M-LRS did not change with time | 7 | 5.63 | 0.58 |
| M-RBF and M-fertility | M-fertility : decade | The association between M-RBF and M-fertility did not change with time | 9 | 7.63 | 0.57 |
|  | M-fertility : M-SES | The association between M-RBF and M-SES was independent of M-SES | 1 | 1.52 | 0.22 |
|  | M-SES : decade | The effect of M-SES on M-RBF did not change with time | 9 | 3.94 | 0.92 |
|  | M-SES | M-RBF did not depend on M-SES when controlling for M-fertility and decade | 1 | 0.085 | 0.77 |
| M-RBF and M-AFR | M-AFR : decade | The association between M-RBF and M-AFR did not change with time | 9 | 14.87 | 0.09 |
|  | M-AFR : M-SES | The association between M-RBF and M-AFR was independent of M-SES | 1 | 0.034 | 0.85 |
|  | M-SES : decade | The effect of M-SES and M-RBF did not change with time | 9 | 2.74 | 0.97 |
|  | M-SES | M-RBF did not depend on M-SES when controlling for M-AFR and decade | 1 | 1.19 | 0.28 |

Note. M-fertility―maternal lifetime fertility; M-AFR―maternal age at first reproduction; decade―the decade when a mother gave her first birth; M-SES―maternal socio-economic status; O-survival―offspring survival rate at age 15; O-breeding―offspring breeding probability; M-LRS―maternal lifetime reproductive success; M-RBF―maternal risk of breeding failure.
